# A 31-year (1990–2020) global gridded population dataset generated by cluster analysis and statistical learning

**DOI:** 10.1038/s41597-024-02913-0

**Published:** 2024-01-24

**Authors:** Luling Liu, Xin Cao, Shijie Li, Na Jie

**Affiliations:** 1 State Key Laboratory of Remote Sensing Science, Faculty of Geographical Science, Beijing Normal University, Beijing, 100875 China; 2 Beijing Engineering Research Center for Global Land Remote Sensing Products, Faculty of Geographical Science, Beijing Normal University, Beijing, 100875 China

**Keywords:** Social anthropology, Geography

## Abstract

Continuously monitoring global population spatial dynamics is crucial for implementing effective policies related to sustainable development, including epidemiology, urban planning, and global inequality. However, existing global gridded population data products lack consistent population estimates, making them unsuitable for time-series analysis. To address this issue, this study designed a data fusion framework based on cluster analysis and statistical learning approaches, which led to the generation of a continuous global gridded population dataset (GlobPOP). The GlobPOP dataset was evaluated through two-tier spatial and temporal validation to demonstrate its accuracy and applicability. The spatial validation results show that the GlobPOP dataset is highly accurate. The temporal validation results also reveal that the GlobPOP dataset performs consistently well across eight representative countries and cities despite their unique population dynamics. With the availability of GlobPOP datasets in both population count and population density formats, researchers and policymakers can leverage the new dataset to conduct time-series analysis of the population and explore the spatial patterns of population development at global, national, and city levels.

## Background & Summary

The world’s population is estimated at over 8 billion and is projected to reach around 8.5 billion by 2030^1^. As population growth continues, the ability to monitor population spatial dynamics over long periods becomes increasingly essential for the implementation of effective policies and initiatives related to sustainable development. Specifically, of the 17 Sustainable Development Goals and 169 targets set by the United Nations^2^ in 2015, approximately half of the indicators require accurate and spatially explicit demographic data. The Sustainable Development Goals emphasize ‘leaving no one behind’, which means we need increasingly spatial-temporal consistent gridded population data to identify areas and groups that are vulnerable to poverty, disease, and other development challenges, enabling more targeted and effective interventions. A continuous gridded population dataset can offer more spatially detailed information and allows for analysis of the unevenly changing relationship between humans and nature at a pixel scale over time. It was recognized as essential data source for various applications, such as epidemiology, urban planning, environmental management, assessment of risks to vulnerable population, energy crises, global inequities, and assessment of progress toward the Sustainable Development Goals (SDGs)^3–10^.

The gridded population data is originally derived from census data, which is typically collected through a formal enumeration, although other methods such as surveys may also be used. After converting the census data table of administrative units or enumeration areas to vector format, it will be reallocated into raster grids^11,12^. Raster grids are a series of cells arranged in rows and columns, where each cell represents a geographic area and contains information about the population within that area. There are two main methods for producing top-down gridded population data: area-weighted and dasymetric mapping, and bottom-up population mapping methods are adopted when census data is not available. Area-weighted mapping assumes that the population is evenly distributed across administrative areas and assigns demographic information to each grid cell based on the proportion of administrative cells covered by each cell. This method is simple and easy to implement but may not accurately reflect the true population distribution, especially in areas with heterogeneous population density^13^. Dasymetric mapping makes assumptions about the relationship between population and various geographic and land cover characteristics and uses ancillary data to determine where and how much population should be assigned to each location. This method may result in more accurate estimates of population distribution, but it requires more detailed ancillary data and expertise to implement.

There are five long time-series of global gridded population data products with either density or count measures, including the Global Human Settlements Layer Population (GHS-POP), the Global Rural Urban Mapping Project (GRUMP), the Gridded Population of the World Version 4 (GPWv4), the LandScan Population datasets and the WorldPop datasets, all with a spatial resolution of 30 arcseconds (about 1 km at the equator). Nonetheless, previous research has identified some limitations associated with these datasets.

First of all, there is currently no continuous long-term gridded population dataset available at a spatial resolution of approximately 1 km, particularly before 2000. Among the three datasets (GHS-POP, GRUMP, and GPWv4), the shortest time interval is five years. Continuous gridded population maps are available after 2000 for the other two datasets (LandScan and WorldPop). However, LandScan’s methods and metadata are updated every year, especially for the 2000s^14^. These products are based on correlations between modeling factors and populations at the administrative unit level and then predicted to gridded populations. Therefore, the accuracy of population spatialization depends on the accuracy of the elements used to a large extent and population allocation methods^8,15^. Besides, there is a mismatch between the training and predicted data under scale variation, resulting in low accuracy of the overall estimate^11,16^.

Secondly, the reliability and uncertainty of population data products are typically described in documentation or validated in specific countries and regions, with methodological and ancillary data uncertainties being the most common sources of uncertainty. Methodological uncertainty issues can arise due to spatial autocorrelation resulting from the equally weighted distribution of the population, leading to overestimation of the population^12,17^. Problems associated with ancillary data include common inaccuracies in land cover data, which typically have an accuracy range of 70–85%^18^. Other ancillary data sources, such as nighttime light data, can also introduce cumulative errors in the gridded population data due to saturation effects, blooming effects, and inter-annual inconsistencies^19^. These errors can undermine the reliability of the ancillary data and propagate into the final population estimates, further increasing uncertainties in the results.

Last but not least, one issue that has received limited attention is the global applicability of gridded population data. The five sets of gridded population data products are used extensively in global-scale studies, but their accuracy and suitability for different regions and situations have not been fully evaluated. Currently, there are ongoing efforts to validate and compare the precision of various population data products, although the findings are frequently restricted to specific countries or regions. For example, Archila Bustos *et al*.^14^ used the example of Sweden, where population change is slow, to validate and compare five demographic datasets with statistical data from 1990–2015, and found that no datasets showed consistent best for different situations, and there were differences in accuracy across datasets in uninhabited areas.

Although population data products are fundamental for many researches and applications, a lack of long-term and consistently highly accurate gridded population data exists for time-series analysis. As assessments of population data product applicability continue to emerge, it has been found that each population data product has its applicability and, in some cases, shows a high degree of accuracy^4,20^. These findings offer insights into the research objective of whether it is possible to integrate these five sets of multi-source demographic data and leverage the strengths of each data through a statistical learning approach to produce a set of new demographic products suitable for long time-series analysis at the global grid scale.

**Fig. 1:**
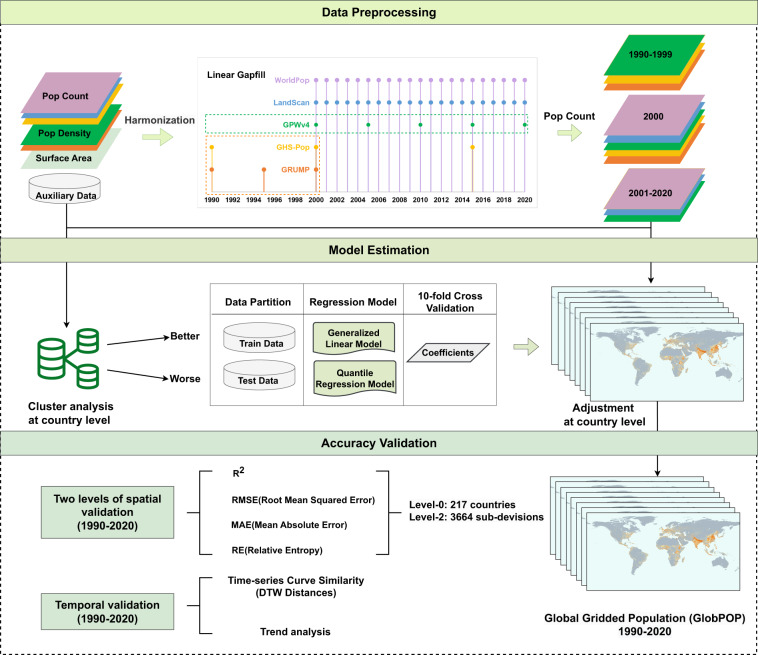
Workflow of the estimation and validation of the global gridded population (GlobPOP).

Hence, this study proposed a data fusion framework to generate a continuous global gridded population (GlobPOP) from 1990 to 2020 using the five existing products. As shown in Fig. 1, the whole framework of population data production is divided into three parts. The first part was pre-processing, which harmonized the data by converting population data format uniformly and linear gap-filling. The second part involved model building and estimation based on cluster analysis and statistical learning. The clustering analysis allowed for understanding the differences in each population dataset’s performance across countries. The estimation model was established through statistical learning and training regression parameters on the regions with better performance. The third part was accuracy validation, which included two levels of spatial and temporal validation. Finally, we examined the model sensitivity and discussed the adaptability of the new data product at pixel scale.

## Methods

In this section, we described the input data and the data fusion framework used in producing the global gridded population data product.

### Materials

This section summarizes the five global population data products used to produce the continuous gridded population. Table 1 shows the detailed information of original input population data sources.

**Table 1:** Information on global population data product datasets utilized to produce continuous gridded population.

Dataset	Unit	Input population source	Resolution (arc-seconds)	Year	Coordinate System	Source URL
GHS-POP	person/pixel	GPWv4.10	30”	1990,2000	WGS-84	https://ghsl.jrc.ec.europa.eu/
GRUMP	person /km^2^	GPWv3	30”	1990,1995, 2000	WGS-84	https://sedac.ciesin.columbia.edu/data/collection/grump-v1
GPWv4.11	person /km^2^	Census	30”	2000,2005,2010,2015,2020	WGS-84	https://sedac.ciesin.columbia.edu/data/collection/gpw-v4
LandScan	person /pixel	Census	30”	2000–2020	WGS-84	https://landscan.ornl.gov/
WorldPop	person /pixel	GPWv4 and Census	30”	2000–2020	WGS-84	http://www.worldpop.org/

GPWv4 is the only dataset that uses area weighting for each year from national census registration data, where a water body mask is first applied before area weighting, to ensure that population is not allocated to water bodies and snow- and ice-covered areas^21^. The limitation lies firstly in the assumption that the population is evenly distributed within administrative boundaries and is, therefore, more accurate for smaller input units than larger ones^22^. Secondly, it can be affected by interpolation, particularly in areas where the population changes dramatically over short periods, leading to population underestimation^23^.

GHS-POP population data are binary dasymetric mapped, with population data derived from the GPWv4 UN-adjusted population dataset at the administrative district level and ancillary data using a gridded dataset of built-up areas, with each grid representing the percentage of cells covered by built-up areas. 95% of the population data is allocated to grid cells in proportion to the density of built-up areas using an area-weighted approach^24^. Only when the administrative district area is less than 250 m grid area, all the population within one grid will be aggregated together, which may lead to a shift in the spatial distribution of population to adjacent grids. As the reallocation of the population in the GHS-POP is based on the density of built-up, which may be allocated to non-residential areas, such as commercial, industrial, and recreational areas, distinguished by the residential population allocated to built-up areas^24^.

The GRUMP data is based on GPWv3 (version 3) to produce improved population gridded data, which redistributes the population to urban and rural areas according to a binary mapping method, with rural and urban areas being divided mainly based on nighttime light data. The GRUMP data refers to the use of nighttime light data such as DMSP, to estimate urban areas where the population is overestimated. Due to the ‘blooming’ effect of nighttime lights, where poorly electrified or un-electrified areas cannot be detected, and therefore the population is underestimated. Moreover, the GPWv3 as the older version is less accurate than GPWv4, and consequently, the GRUMP data is less accurate than GPWv4 in some regions^12^.

LandScan data uses multivariate mapping to assign local census data to each grid cell according to the likelihood coefficient between the auxiliary data and the population. As the metric values represent integer counts of the environmental population, which is the average population for a typical 24-hour day, week, and season, and therefore also reflect the distribution of the working, and traveling population, such as in urban areas where there is a problem of population overestimation. The LandScan algorithm is updated annually to introduce more and higher precision data, which is not conducive to time-series comparisons of LandScan data, as changes can be caused not only by population changes but also by changes in input data or algorithms^25^.

A random forest model is employed in the WorldPop data production process to generate population projections based on ancillary data such as land cover, elevation, nighttime lights, roads, and settlements. Population input data from census and official population estimation databases linked to GIS through the WorldPop initiative and built on GPWv4 are then assigned to each country/region based on population projections^13^. The random forest projections in the WorldPop data do not exceed the input population range.

Besides the gridded population data, we used some other ancillary data as well. The vector boundary shapefiles were utilized for zonal statistics at two scales, and census data were used for cluster analysis and model validation. Since census data is still considered more accurate and reliable compared to gridded population data, the country administrative level census data as reference data was used to explore where are the better regions for various gridded population data products in different years. Meanwhile, we also employed the two spatial scales (level-0 is the country administrative level, and level-2 is the sub-division of the subnational administrative level) to validate the results and for sensitivity analysis. Furthermore, the surface area layer was exploited for population density calculation. The detailed information is displayed in Supplementary Table 1.

GADM, or Database of Global Administrative Areas, is a highly accurate global database of administrative boundaries. As we performed the zonal statistics at two levels, we only use these two levels’ boundary shapefiles. For level-0 boundaries, we matched the ISO country code with census data and acquired the 217 countries’ boundaries. And for level-2 boundaries, we chose the nine countries’ level-2 administrative units across five continents (Asia, Europe, America, Africa, and Oceania), which were processed and harmonized to match the definitions used in the level-2 census data from 1990 to 2020.

The census data provides detailed information on the population size, age structure, and geographic distribution of a specific area. For the level-0 census data, the World Population Prospect (WPP) 2022^1^ provides population estimates and projections for countries and regions worldwide. In this study, only the population estimates for countries from 1990 to 2020 were considered for two aspects. On the one hand, the WPP was used as reference data in cluster analysis to explore where the better regions are for various gridded population data products in different years, which helped to improve the accuracy of the population estimates. On the other hand, it was of great significance to validate the results’ spatial-temporal consistency for 217 countries from 1990 to 2020. In addition, we collected level-2 census data from nine countries across five continents, including China and India in Asia, the United Kingdom in Europe, the United States in North America, South Africa, Nigeria, and Angola in Africa, and New Zealand and Vanuatu in Oceania. These data covered the period from 1990 to 2020 and were obtained from each country’s bureau of official statistics.

### Data preprocessing

The data preprocessing consists of two steps, data harmonization and linear gapfill.

#### Data harmonization

The harmonization process includes the raster data conversions and census data regulations. We converted the input population density products to population count layers, by overlaying the surface area layer. Because the population count data are originally in a geographic coordinate system, the closer the grids get to the Poles, the more they become narrower and smaller. This holds even after the polygons are projected, it is more accurate to calculate raster algebra. What’s more, we excluded some uninhabited countries, island countries and regions in the census data as Supplementary Table 4 shows, and finally acquired census data of 217 countries with matched names.

#### Linear gapfill

Considering the gaps in different population data products are between five to ten years, we took the linear population growth assumptions to fill the data gaps. The linear gapfill process included linear interpolation and extrapolation at the pixel level. The linear interpolation formula is as in Eq. (1):

1 $$y={y}_{1}+{\rm{(}}{y}_{2}-{y}_{1}{\rm{)}}\cdot \frac{t-{t}_{1}}{{t}_{2}-{t}_{1}}$$

where *y* signifies the estimated population at a specific time, *y*_1_ corresponds to the population at the first known time, *y*_2_ denotes the population at the second known time, *t* represents the target time for which we want to estimate the population, *t*_1_ is the time of the first known population value, *t*_2_ is the time of the second known population value. This formula is essentially a linear interpolation formula. It calculates the population at a particular time *t* by considering the linear growth between the known population values (*y*_1_ and *y*_2_) at the times *t*_1_ and *t*_2_.

The data interval is usually 5 years, if data is not available within 5 years, 10 years interval is used. Thus, the five products are divided into three parts as shown in the top position of Fig. 1. From 1990 to 1999, we performed the linear interpolation and extrapolation for GHS-POP, GRUMP, and GPWv4. For the year 2000, we kept the data for all five original population data products. And from the year 2001 to the year 2020 we carried out the linear interpolation for the GPWv4.

### Model estimation

The key point of the data fusion framework is to fully comprehend and exploit the strengths and weaknesses of the five input population data products, contributing them to the regression model of population fusion. Thus, this study performed the clustering analysis which allowed for understanding the differences in each population dataset’s performance across countries. And then the estimation model was established through statistical learning and training regression parameters on the regions with better performance.

#### Cluster analysis for spatial consistency

Cluster analysis is an unsupervised approach, and the most common method is the K-means cluster method^26^. The statistical software used for cluster analysis is RStudio, and the packages include’cluster’,’quantreg’ and’Metrics’. Clustering allows for the identification and categorization of homogeneous groups of the dataset. Four metrics were selected to quantify the similarity between actual census and product population counts at the country level. And we used these differences to identify areas with less variation for population projections.

First of all, we selected the *APE (Absolute Percentage Error), SE (Squared Error), SLE (Squared Logarithmic Error), and Dif (Difference) indexes* to compare different population data products with census data. These indexes were chosen to facilitate a comprehensive comparison between different population data products and the corresponding census data in cluster analysis.

2 $$APE=\frac{{X}_{i}-{Y}_{i}}{{X}_{i}}$$

3 $$SE={({X}_{i}-{Y}_{i})}^{2}$$

4 $$SLE={\rm{(ln}}\,{\rm{(1+}}{X}_{i}{\rm{)}}-{\rm{ln}}\,{\rm{(1+}}{Y}_{i}{{\rm{))}}}^{{\rm{2}}}$$

5 $$Dif=({Y}_{i}-{X}_{i})$$

where the *X*_*i*_ is the actual value of population count, and the *Y*_*i*_ is the predicted value of population count.

Then the data were scaled to a standard range, between 0 and 1, to remove any potential bias that might be introduced by different measurement scales. Thirdly, we determined the ideal number of clusters for the datasets and performed K-means clustering analysis. It involves iteratively assigning data points to different clusters based on their similarity and calculating the centroids of each cluster. Finally, the country-level census data were divided into 2 categories. The better product data which have higher similarities with census data will be utilized for model parameters training, and the worse will take part in model parameters testing.

#### Model estimation

To train regression parameters for population fusion based on countries with better performance, we selected two statistical regression models for population prediction. Regression methods such as the generalized linear model (GLM) and quantile regression model (QRM) can be effective in controlling for confounding factors in a research study^27^. The generalized linear model (GLM) is an extension of the linear regression model that extends the possible distribution of residuals to a family of distributions called the exponential family, allowing the dependent variable to be non-normal^28^. In GLM, the confounding factors can be included as covariates in the model, along with the independent variables of interest. The coefficients for the independent variables can then be estimated while controlling for the effects of the confounding factors. The quantile regression model (QRM) is more efficient and robust to outliers^29^. In QRM, the focus is on estimating the conditional quantiles of the dependent variable, rather than the mean. This can be useful when the relationship between the independent and dependent variables is not well approximated by a linear relationship. QRM can also be used to estimate the conditional quantiles while controlling for the effects of the confounding factors. The GLM and QRM can both be expressed as given below:

6 $${Y}_{t}={a}_{1,t}{X}_{1,t}+{a}_{2,t}{X}_{2,t}+\ldots +{a}_{n,t}{X}_{n,t}+{\varepsilon }_{t}$$

where *Y*_*t*_ is the predicted population of the target t year, *X*_*n,t*_ is the n available population data product in the target t year, and *a*_*n,t*_ is the weight coefficient of the n available population data product in the target *t* year.

Given that population counts should inherently be non-negative, we employ the L-BFGS-B (Limited-memory Broyden–Fletcher–Goldfarb–Shanno Bound-constrained) algorithm for parameter estimation within the model. The algorithm is a well-established optimization technique, often used in constrained optimization problems^30^. Specifically, we impose lower bounds on the estimated coefficients to ensure their non-negativity.

We trained the two regression models at the national level to obtain the parameters needed for the production of population data product. The model output was used as coefficients of linear regression prediction at the pixel scale. During the training process, we took 10-fold cross-validation and 200 iterations on average to obtain the optimal parameters.

#### Population adjustment

For quality control, two steps are carried out to ensure the reliability of GlobPOP dataset. We took the UN World Population Prospects 2022 as a reference standard, with the model projections for each country adjusted to the UN agencies’ generic global national population statistics. We applied the adjustment to 217 countries, excluding uninhabited islands and territories.

Adjustment factors for matching national estimates to UN estimates:

7 $${a}_{t}=\frac{{P}_{un,t}}{\sum {P}_{x,t}}$$

where a_t_ is the adjustment factor in the target year, *P*_*x,t*_ is the pixel population count in the target year within the national administrative region, and *P*_*un,t*_ is the UN national estimate for the target year.

Adjustment factors were applied at the pixel level within each country boundary:

8 $${P}_{adj,t}={P}_{x,t}\times {a}_{t}$$

where *P*_*adj*,*t*_ is the sub-national UN WPP-adjusted estimate, and *P*_*x,t*_ and *a*_*t*_ are as defined in Eq. (7).

Furthermore, the projected population for each year will be evaluated to determine if they are below zero. If this is the case, they will be adjusted to zero to ensure that negative population numbers are not recorded.

### Accuracy validation

To scan the GlobPOP products fully and thoroughly, we employed the validation in three aspects. Table 2 shows the accuracy indexes and their equation definitions for spatial and temporal validation in this study.

**Table 2:** Model accuracy metrics calculated in this study.

Metrics	Equation	Description
R^2^	${{\rm{R}}}^{{\rm{2}}}=1-\frac{{\sum }_{i=1}^{n}{({x}_{i}-{y}_{i})}^{2}}{{\sum }_{i=1}^{n}{(\bar{x}-{x}_{i})}^{2}}$	Higher values indicate a better fit.
RMSE	$\mathrm{RMSE}=\sqrt{\frac{1}{n}\mathop{\sum }\limits_{i=1}^{n}{({x}_{i}-{y}_{i})}^{2}}$	Lower values indicate a better fit.
MAE	$\mathrm{MAE}=\frac{1}{n}\mathop{\sum }\limits_{i=1}^{n}| {x}_{i}-{y}_{i}|$	Lower values indicate a better fit.
RE	$RE=\underset{-\infty }{\overset{\infty }{\int }}P(x)\cdot \log (\frac{P(x)}{Q(y)})dx$	Higher values indicate a better fit.
DTW distance	Warping curve: $\phi (t)=({\phi }_{x}(t),{\phi }_{y}(t))$, t = 1,…,T ${d}_{\phi }(X,Y)=\mathop{\sum }\limits_{t=1}^{T}d({\phi }_{x}(t),{\phi }_{y}(t)){m}_{\phi }(t)/{M}_{\phi }$ DTW distance: $D\left(X,Y\right)=\min \,{d}_{\phi }\left(X,Y\right)$	Lower values indicate a better fit.

Note: x is the census data, y is the predicted data, ${m}_{\phi }\left(t\right)$ is a per-step weighting coefficient and ${M}_{\phi }$ is the corresponding normalization constant.

For spatial validation, we used four indicators (R^2^, RMSE, MAE, and Relative Entropy) to explore the overall accuracy in 217 countries and nine countries’ level-2 regions. The metric R square (R^2^) represents the proportion of variance in the dependent variable, which describes the extent to which the variance of one variable explains the variance of a second variable. The Root Mean Squared Error (RMSE) is a common measure of the quality of the model fit. The Mean Absolute Error (MAE) is also a common measure of the error between pairs of observations of the same phenomenon. In addition to relative entropy (RE), the metric is used to measure the probability distribution difference between the predicted population count and census data.

As for the temporal validation, the time-series curve similarities and trend analysis were taken into consideration. We chose eight countries and their most populated or capital cities, and performed the temporal validation at two levels. The Dynamic Time Warping (DTW) distances method is a normal and popular method to measure the time-series curve similarities^31^. It aims to find the minimal distance between two time-series curves.

The Sen’s slope estimator and non-parametric Mann-Kendall test are widely used in the long time-series trend analysis for many fields, such as meteorology^32–34^. The Mann-Kendall test statistic can be expressed as given below:

9 $$sgn\left(x\right)=\left\{\begin{array}{c}+1,\,if\,x > 0\\ 0,\,if\,x=0\\ -1,\,if\,x < 0\end{array}\right.$$

10 $$S=\mathop{\sum }\limits_{k}^{n-1}\mathop{\sum }\limits_{j=k+1}^{n}sgn\left({X}_{j}-{X}_{k}\right)$$

11 $${Z}_{s}=\left\{\begin{array}{c}\frac{S-1}{\sqrt{Var\left(S\right)}},\,if\,S > 0\\ 0,\,if\,S=0\\ \frac{S+1}{\sqrt{Var\left(S\right)}},\,if\,S < 0\end{array}\right.$$

where *X*_*j*_ and *X*_*k*_ are the sequential data values, *n* is the length of the data, *Z*_*s*_ is the normalized test statistics.

And then Sen’s slope estimator can be calculated using Eqs. (12) and (13).

12 $${d}_{k}=\frac{{X}_{j}-{X}_{i}}{j-i},\,{\rm{for}}\,i=1,\,\ldots ,\,{\rm{N}}.$$

13 $$Sen=Median\left({d}_{k}\right)$$

where *d*_*k*_ is the value of the slope, and *Sen* is the Sen’s slope estimator.

## Data Records

The continuous global gridded population data product^35^ (GlobPOP 1990–2020) in the WGS84 coordinate system with a spatial resolution of 30 arcseconds (approximately 1 km in equator) can be freely accessed on Zenodo at 10.5281/zenodo.10088105. The data is stored in the GeoTIFF format for each year. There are two population formats available: ‘Count’(Population count per grid) and ‘Density’(Population count per square kilometer each grid). The current version of the product covers the globe from 90 N latitude to 90 S.

Each GeoTIFF filename has 5 fields that are separated by an underscore “_”. A filename extension follows these fields. The fields are described below with the example filename: GlobPOP_Count_30arc_1990_I32.

Field 1: GlobPOP(Global gridded population)

Field 2: Pixel unit is population “Count” or population “Density”

Field 3: Spatial resolution is 30 arc seconds

Field 4: Year “1990”

Field 5: Data type is I32(Int 32) or F32(Float32)

## Technical Validation

### Cluster results

**Fig. 2:**
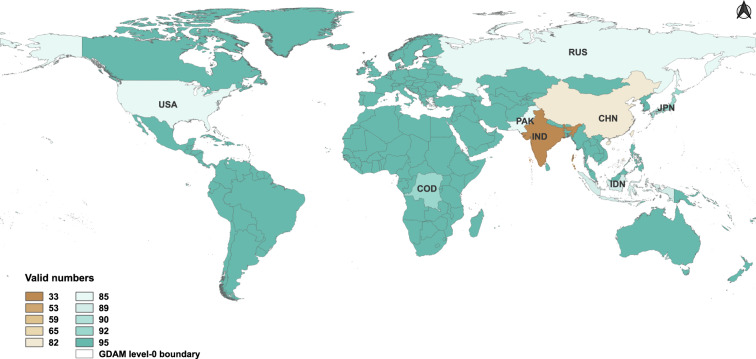
The number of valid sets of population data products for 217 countries from 1990 to 2020.

The cluster analysis was performed to quantify the accountability of the current five global gridded population data products, which is represented by the similarity between actual census and product population counts at the country level. In Fig. 2 and Supplementary Table 2, we provided explicit information on which global gridded population data products are not valid in a specific year for different countries, and that can guide the users on whether or not they should use these products in the study area of interest. Figure 2 shows that the numbers for which the population data products are accountable are distributed unevenly in all 217 countries for the past three decades. It quantifies the accountability of these data products by indicating how many of them can be trusted for each country in a given year. As observed in the Fig. 2, the numbers vary across countries and years. The uneven distribution of valid data sets highlights that the reliability of these products fluctuates over time and is not uniform across all regions.

The greater the valid numbers are, the more product data get involved in the following model training procedures. The top three countries with the lowest number of active products are India, Guadeloupe, and the Republic of Maldives. In total, 12 countries show that no less than one product set is unreliable for one or more of the past years 1990–2020.

### Spatial accuracy validation

#### Level-0 accuracy

The findings of this study reveal that GlobPOP has a high level of accuracy in predicting country-level population estimates, shown in Table 3. The overall R^2^ of GlobPOP is greater than 0.999 when compared with the World Population Prospects 2022. The range of Root Mean Squared Error (RMSE) values observed was between 120423 and 296066, while the Mean Absolute Error (MAE) values ranged from 48243 to 84103. Additionally, the largest relative entropy was less than 0.1. During the model estimation process, the quantile regression model (QRM) exhibited stable performance and outperformed the general linear model (GLM) tested in terms of both predictive accuracy and consistency. Therefore, we selected the QRM as the population prediction model.

**Table 3:** Accuracy metrics at level-0 scale from 1990 to 2020.

Year	RMSE	MAE	Relative entropy
1990	120,423.353	48,243.115	0.008
1991	256,783.525	72,354.328	0.038
1992	257,177.260	73,229.177	0.035
1993	255,930.564	74,003.526	0.035
1994	137,396.778	50,624.452	0.015
1995	259,304.072	75,973.255	0.035
1996	270,164.279	77,203.126	0.037
1997	274,992.760	78,344.594	0.039
1998	280,552.406	79,361.923	0.043
1999	285,944.660	80,456.476	0.042
2000	246,056.942	71,651.535	0.047
2001	223,478.015	63,369.909	0.038
2002	223,469.611	63,024.679	0.025
2003	236,657.490	66,128.909	0.035
2004	237,735.200	66,803.389	0.035
2005	240,226.741	68,510.560	0.028
2006	236,346.429	67,497.998	0.028
2007	246,290.367	70,017.576	0.037
2008	157,570.549	50,698.258	0.011
2009	155,296.131	52,465.263	0.008
2010	249,214.418	71,935.985	0.052
2011	257,686.207	74,504.449	0.063
2012	263,606.439	75,997.545	0.057
2013	269,468.057	77,288.322	0.066
2014	279,812.049	79,058.192	0.086
2015	296,066.337	84,102.538	0.070
2016	288,390.994	83,084.648	0.052
2017	166,830.045	54,751.908	0.016
2018	132,079.934	51,094.696	0.019
2019	144,898.231	52,834.512	0.025
2020	172,456.799	58,858.456	0.021
Average accuracy	229,751.827	68,176.558	0.037

#### Level-2 accuracy

Table 4 demonstrates that the average *R*^*2*^ is higher than 0.972 for all census available countries at the level-2 scale when compared with the corresponding level-2 census data. The range of Root Mean Squared Error (RMSE) values observed was between 11158 and 272229, while the Mean Absolute Error (MAE) values ranged from 3065 to 49844. Moreover, the mean relative entropy was less than 3.406. These findings highlight the strong performance and accuracy of the population prediction model at the level-2 scale.

**Table 4:** Accuracy metrics at level-2 scale from 1990 to 2020.

Year	R^2^	RMSE	MAE	Relative Entropy
1990	0.996	19,409.489	5,971.134	4.051
1991	0.910	185,423.919	28,184.222	9.872
1996	0.979	41,881.703	10,069.021	12.427
2000	0.996	89,359.155	16,212.022	0.670
2001	0.918	228,714.182	31,130.438	1.486
2006	0.991	26,268.296	7,162.628	1.623
2009	1.000	8,728.191	2,547.121	0.835
2010	0.992	140,002.895	25,753.600	0.566
2011	0.916	272,229.082	32,903.260	1.189
2014	0.999	12,424.848	4,008.583	1.614
2016	0.999	14,655.628	4,212.231	1.889
2020	0.978	259,976.075	48,883.768	1.262
Average accuracy	0.973	108,256.122	18,086.502	3.124

### Temporal accuracy validation

#### Country-level accuracy

**Fig. 3:**
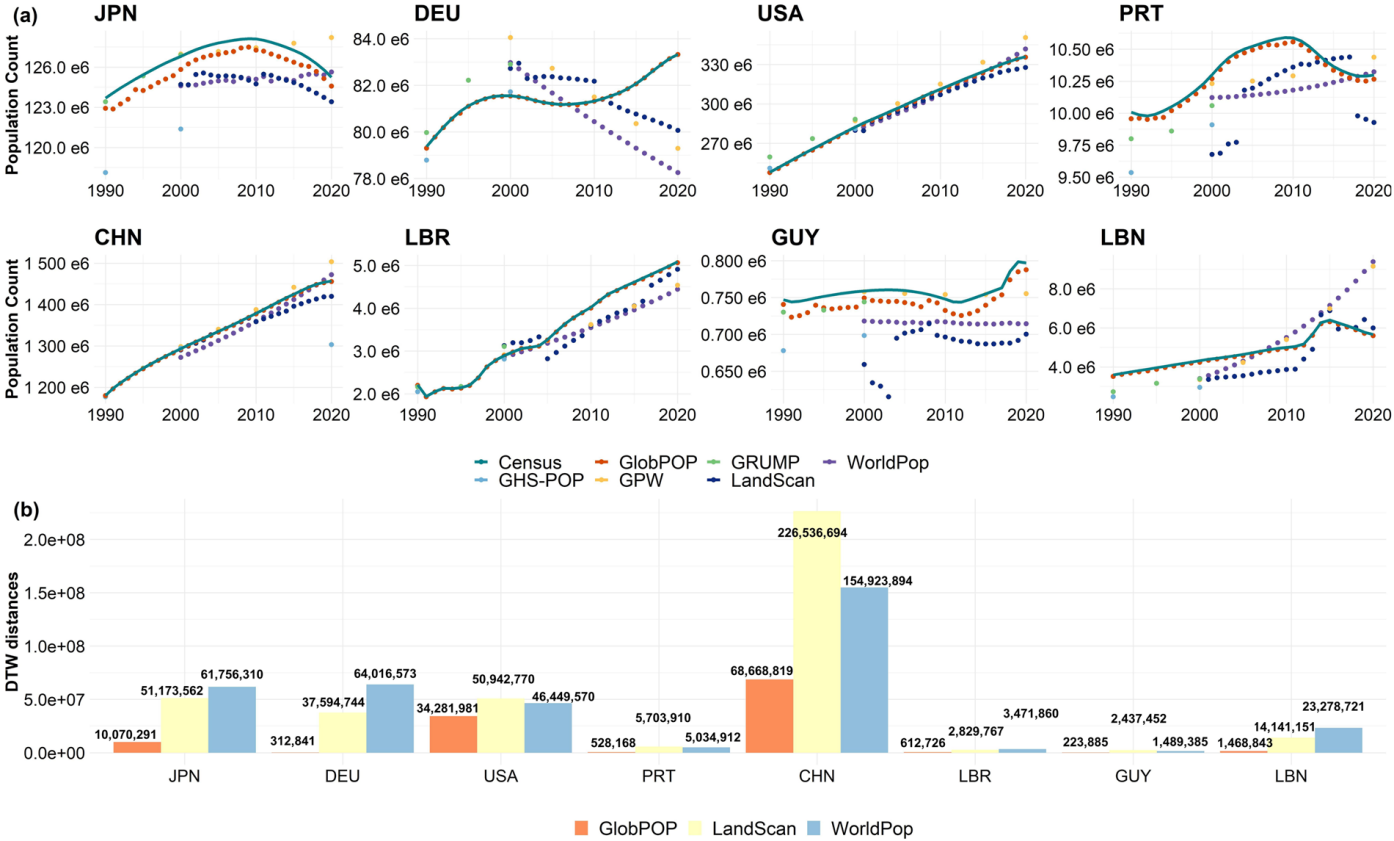
Comparison of the GlobPOP and the other datasets over the eight countries. (**a**)The population count time-series curve in eight countries from 1990 to 2020. (**b**)The population time-series curve DTW distances of the GlobPOP, LandScan, and WorldPop datasets in eight countries from 2000 to 2020.

To validate the temporal accuracy of GlobPOP at the country level, we randomly selected eight countries from five different continents, consisting of four developed countries (Japan (JPN), German (DEU), United States (USA), Portugal (PRT)) and four developing countries (China (CHN), Liberia (LBR), Guyana (GUY), Lebanese Republic (LBN)). These countries were chosen due to their distinct population trends, representing a diverse range of demographic and socioeconomic characteristics. We compared the population counts time-series curves of the GlobPOP dataset with the other five available datasets, from 1990 to 2020. The results are presented in Fig. 3(a). In the developed countries, the GlobPOP dataset shows the most consistent curve variations with the census curve, while the other dataset shows obvious disparity with census curve especially in Germany.

It is worth mentioning that there are slight differences between the curves for Japan and Guyana in Fig. 3(a), even though the curves’ trends are matched. This is due to the method used to calculate the national adjustment factor, which is rasterized from a vector file. For small countries with long coastlines, some of the small pixels were excluded during the rasterization process, which resulted in a curve that is not the same as the census data curve. This issue may have implications for the accuracy of the population estimates in these small countries, especially at a finer spatial resolution. To address this issue, our future studies will explore alternative methods for calculating the national adjustment factor that takes into account the specific characteristics of small countries with long coastlines. Nonetheless, the overall results of this study suggest that the population estimation models and products evaluated in this study could be useful for generating reliable population data at different spatial scales.

Furthermore, we computed the Dynamic Time Warping (DTW) distances between the population time-series curves of the three datasets from 2000 to 2020 in the same eight countries. The DTW distances represent the similarity between two time-series curves, with smaller distances indicating higher similarity. As presented in Fig. 3(b), GlobPOP’s DTW distances are the smallest in the eight countries. For example, the GlobPOP dataset outperforms the other dataset in Guyana and Lebanese Republic, the DTW distances of WorldPop and LandScan are statistically six times larger than GlobPOP. The results display a large disparity of population change from 2000 to 2020 for WorldPop and LandScan comparing with census data in both countries. These comparisons provide evidence of the high temporal accuracy of the GlobPOP dataset, which consistently outperforms the other datasets tested across all eight countries, regardless of whether the countries were classified as developed or developing.

#### City-level accuracy

**Fig. 4:**
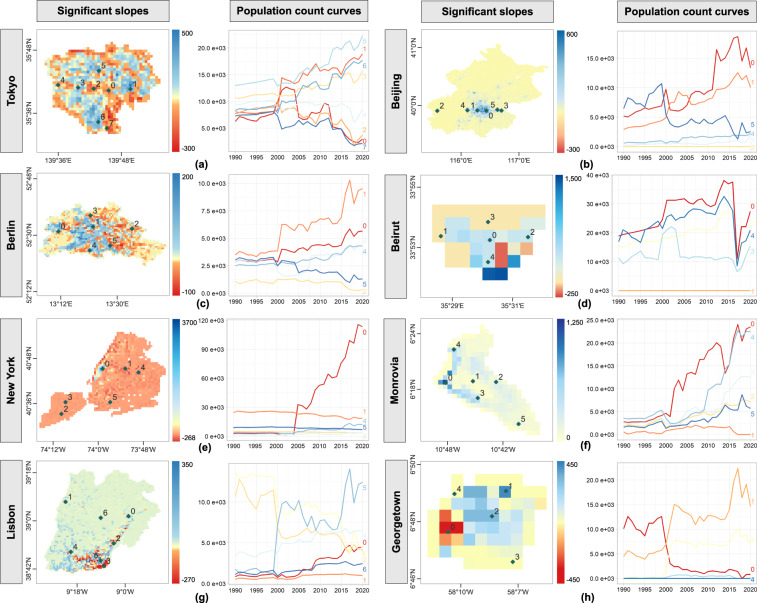
The temporal population trend analysis with significant slopes and pixel population curve variations in eight cities. (**a**) Tokyo in Japan. (**b**) Beijing in China. (**c**) Berlin in German. (**d**) Beirut in the Lebanese Republic. (**e**) New York in the United States. (**f**) Monrovia in Liberia. (**g**) Lisbon in Portugal. (**h**) Georgetown in Guyana.

More importantly, to validate the temporal accuracy of GlobPOP at the city level, we focused on the most populated or capital cities of the above eight countries. Through trend analysis and exploration of pixel population count curve variations, we aimed to examine the GlobPOP dataset’s performance in capturing population dynamics at the local scale. Specifically, Fig. 4(a),(c),(e),(g) presents the pixel population count curves with both positive and negative slopes, with the curve trends consistently aligned with the trend analysis results.

Nonetheless, in the cities of developing countries, as Fig. 4(b),(d),(f),(h) shows, the curve fluctuations of pixels are significantly different, particularly in smaller cities, such as Beirut in Fig. 4(d). where there is a clear discontinuity in pixels showing significant growth or decline trends from 2015 to 2020. This phenomenon is caused by the fact that the QRM model assigned more weight to LandScan since 2016, making the population distribution of GlobPOP data more similar to that of LandScan. As the LandScan data is defined as a nighttime population rather than the residential population, LandScan is more realistic in terms of spatial detail, but it is fundamentally different from other population data products. As a result, the spatial distribution of GlobPOP over the last five years and at a finer scale is somewhat inconsistent with what it was before, and further calibration is needed to adjust the parameters of the model.

### Spatial distributions

**Fig. 5:**
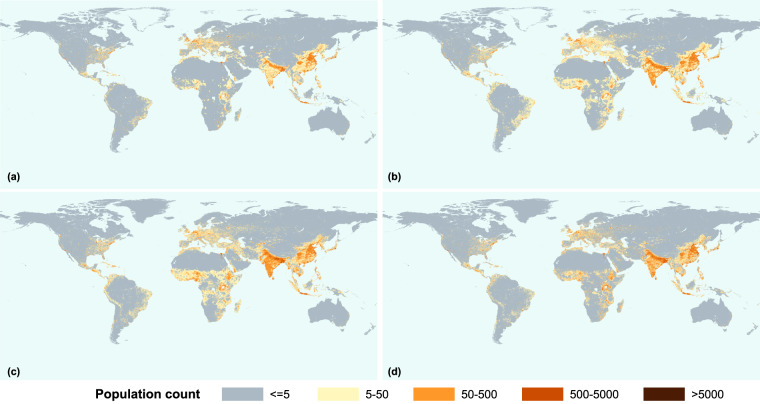
The global gridded population distribution from 1990 to 2020. (**a**)Global population distribution in 1990. (**b**)Global population distribution in 2000. (**c**)Global population distribution in 2010. (**d**)Global population distribution in 2020.

Figure 5 provides a comprehensive overview of global population development over the past three decades. The pixel with population higher than 5,000 has increased significantly in India, China, western Europe, the eastern and southern United States, and South Sahara Africa since 1990. As Fig. 5(d) shows, the pixels with population count range from five to fifty diminish and instead the pixels with population no larger than five increased, it looks like the population has decreased in these areas. The observed phenomenon can be attributed to the changes in the weighting of the QRM model towards LandScan since 2016 as Supplementary Table 3 shown. This has resulted in a greater resemblance between the population distribution of GlobPOP and LandScan datasets. While LandScan provides a more detailed representation of nighttime population, it differs significantly from other population data products due to its nature of being defined as nighttime population rather than residential population. Consequently, there exists a certain degree of inconsistency in the spatial distribution of GlobPOP at a finer scale over the past five years as compared to previous years. Further calibration of the model parameters which is necessary to reconcile this disparity will be considered in the next following work.

### Benchmark test

**Fig. 6:**
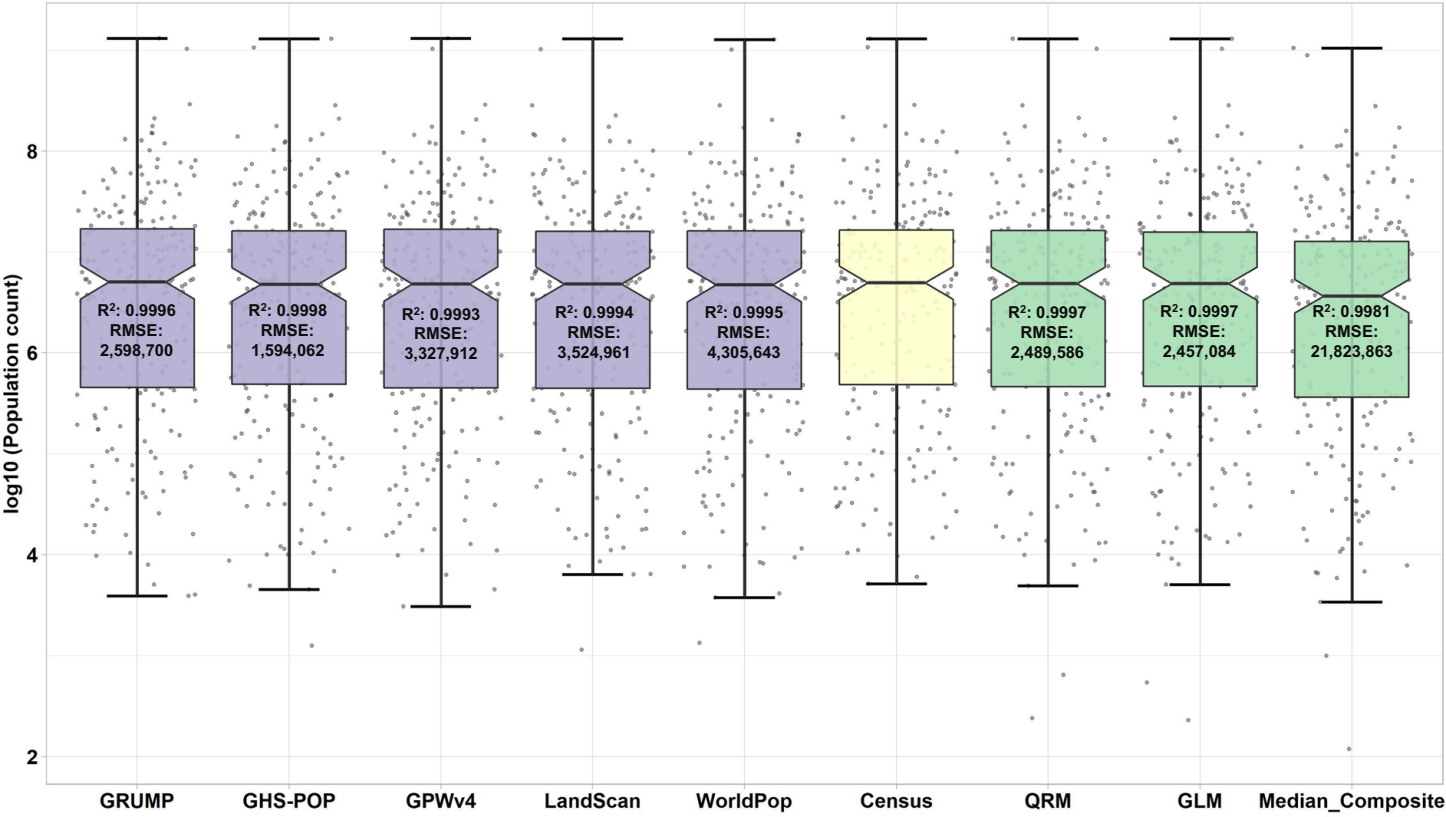
Level-0 population count notched boxplots with data points after log10 transformation, and accuracy comparisons for five population data products and three different population prediction models in 2000.

**Fig. 7:**
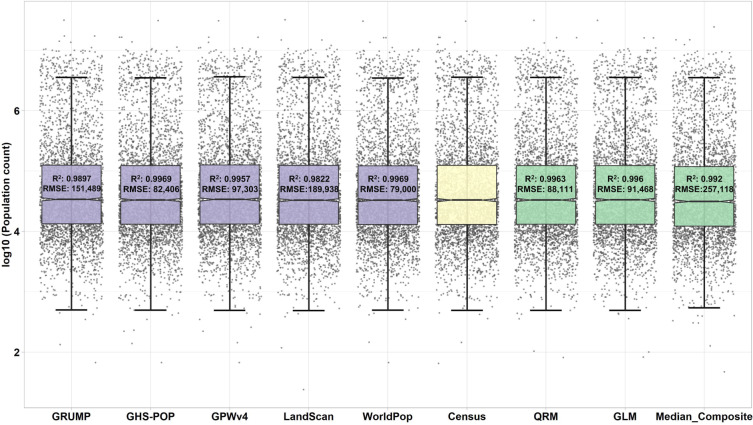
Level-2 population count notched boxplots with data points after log10 transformation, and accuracy comparisons for five population data products and three different population prediction models in 2000.

A benchmark test was performed to evaluate the performance of three population fusion models, namely QRM, GLM, and Median-composite model, along with five global gridded population data products. The objective was to compare the models and population data products for the year 2000, which was the only year when all five datasets were available in their entirety. Other years were unsuitable for benchmarking tests as the population data products were interpolated. Figures 6 and 7 display the population count scatter plot after log10 transformation and accuracy comparisons for the five population data products and the three different model predicted populations at level-0 and level-2 scales, respectively. The results show that the QRM model performed better than the other two models at a finer scale, with an R-squared value of 0.9963. The QRM model maintains high accuracy at the level-0 scale as well, with an R-square value of 0.9997, which is similar to the performance of the GLM model. Based on these results, the QRM model was selected as the final population estimation model for this study.

In summary, the QRM model demonstrates the best performance among the three population fusion models and the existing five population data products. The high accuracy of the QRM model at the level-0 scale also makes it a reliable choice for population estimation.

**Fig. 8:**
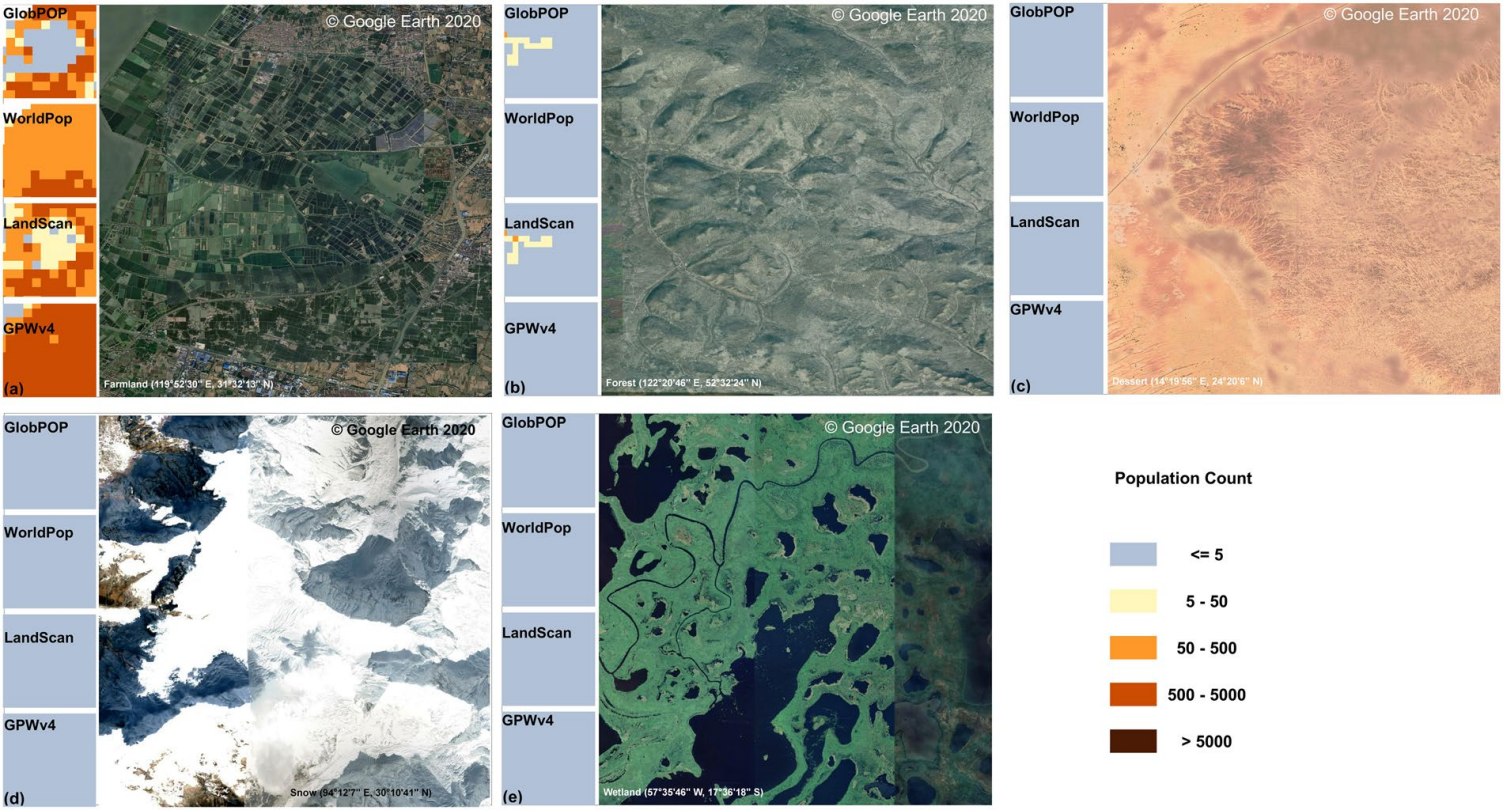
Examples of population distribution at pixel level and the google earth image in 2020. (**a**) Farmland in western China. (**b**)Forest in northern China. (**c**) The Sahara Desert in Africa. (**d**) Snow mountain in west-eastern China. (5) Pantanal wetland in South America.

With the spatial resolution at 30 arc-second, GlobPOP provides more detailed population distribution than conventional census data. The spatial validation results demonstrate the effectiveness of the GlobPOP model in generating reliable and precise population estimates at level-0 and level-2 scales. We also investigated the accountability of GlobPOP to estimate population in the rarely populated land cover areas at pixel scale, five different land cover types (cropland, forest, wetland, desert, and snow) were selected to test the data. As Fig. 8 shown, GlobPOP performs better in capturing population distribution in cropland compared to other products, while its performance is equivalent to other products in other land cover types. Since the real land surface data are not available, and the land cover/use products typically have its uncertainty and bias. There is a lack of reference data to perform spatial validation for gridded population data at pixel level. The selected sample areas include five different land cover types, and we believe the visual inspection could show the accountability of GlobPOP to some degree.

What’s more, to analyze changes in population distributions and for long time-series analysis, a data product constructed from data layers representing the relevant period would be preferred. But there is no global gridded population dataset at approximately 1 km for the past three decades. The temporal validation results demonstrate that the GlobPOP dataset performs consistently well across all eight countries, despite their unique population dynamics. And GlobPOP dataset’s performance in capturing population dynamics at the local scale is also proven. The two-level temporal validation underscores the reliability and versatility of the population prediction model in generating accurate and consistent population estimates over time. Nonetheless, we are obliged to emphasize the disparity of the GlobPOP dataset before and after 2016. The regression model relies on coefficients trained from cluster results, as assigned more weights to LandScan since 2016. Further calibration of the model parameters which is necessary to reconcile this disparity will be considered in the following work.

## Usage Notes

The input datasets and census data are all available on their official website^36–41^. The programs used to generate and validate the gridded population dataset were GRASS GIS (8.2), Python(3.9) and RStuido (2022.07.2). The zonal statistics were performed at QGIS (3.22). All software needs to be installed in Windows 10.

### Supplementary information

Supplementary Information

## Data Availability

The fully reproducible codes are publicly available at GitHub (https://github.com/lulingliu/GlobPOP).
