# Supplementary material for: A 31-year (1990–2020) global gridded population dataset generated by cluster analysis and statistical learning
Source: Sci Data. 2024 Jan 24;11:124. doi: 10.1038/s41597-024-02913-0 (PMC10808219; doi:10.1038/s41597-024-02913-0)
Supplement: Supplementary file 1 — Supplementary Information [file 41597_2024_2913_MOESM1_ESM.docx]

### Additional Information

**Supplementary Table 1. Detailed information of the auxiliary data used in this study**

**Supplementary Table 2. The worse cluster results of five available global gridded population data products at the country level 1990-2020**

**Supplementary Table 3. The Coefficients of the quantile regression model for population prediction 1990-2020**

**Supplementary Table 4. The countries of census data that were excluded of model validation**

**Supplementary Table 1. Detailed information of the auxiliary data used in this study**

| Data | Scale | Year | Source |
| --- | --- | --- | --- |
| GDAM Shapefile | - | 2018 | https://gadm.org/download_world40.html |
| Surface Area: GPWv4 land_water_area.tif | 30’’ | 2019 | https://sedac.ciesin.columbia.edu/data/collection/gpw-v4 |
| World Pop Prospect | 217 Country Administration | 2022 | https://population.un.org/wpp2022-embargoed/ |
| India Census | Level-2 Administration | 1991,2001,2011 | https://censusindia.gov.in/census.website/data/census-tables# |
| China Census | Level-2 Administration | 2000,2010,2020 | https://www.citypopulation.de/en/china/admin/ |
| Angola Census | Level-2 Administration | 2014 | https://www.ine.gov.ao/inicio/estatisticas  https://www.citypopulation.de/en/angola/admin/ |
| South Africa Census | Level-2 Administration | 1996,2001,2011,2016 | https://www.citypopulation.de/en/southafrica/admin/ |
| Nigeria Census | Level-2 Administration | 1991,2006 | https://nigeria.opendataforafrica.org/xspplpb/nigeria-census |
| Vanuatu Census | Level-2 Administration | 2009,2016 | https://www.citypopulation.de/en/vanuatu/admin/ |
| New Zealand Census | Level-2 Administration | 1996,2001,2006 | https://www.citypopulation.de/en/newzealand/admin/ |
| UK Census | Level-2 Administration | 1991-2020 | https://www.nomisweb.co.uk/query/select/getdatasetbytheme.asp?theme=32 |
| US Census | Level-2 Administration | 1990-2020 | https://www.census.gov/data/tables/time-series/demo/popest/estimates-and-change-1990-2000.html  https://www.census.gov/data/datasets/time-series/demo/popest/intercensal-2000-2010-counties.html  https://www.census.gov/programs-surveys/popest/technical-documentation/research/evaluation-estimates/2020-evaluation-estimates/2010s-counties-total.html |

**Supplementary Table 2**. **The worse cluster results of five available global gridded population data products at the country level 1990-2020**

| Year | Grump | | GPW | | | GHS | | | |
| --- | --- | --- | --- | --- | --- | --- | --- | --- | --- |
| 1990 | India, Guadeloupe, Netherlands Antilles | | Russian Federation, United States | | | Indonesia, Japan, Pakistan, Maldives, Swaziland, Guadeloupe | | | |
| 1991 | China, India, Guadeloupe | | Russian Federation, United States | | | Indonesia, Japan, Pakistan, China, Swaziland, Maldives, Guadeloupe | | | |
| 1992 | China, India | | Russian Federation, United States | | | Pakistan, China | | | |
| 1993 | Guadeloupe | | Russian Federation, United States | | | China, Pakistan, Swaziland, Maldives, Guadeloupe | | | |
| 1994 | India | | Russian Federation, United States | | | China, Pakistan, Japan, Indonesia, Swaziland, Maldives, Guadeloupe | | | |
| 1995 | India | | Russian Federation, United States | | | Indonesia, India, China, Pakistan | | | |
| 1996 | China, India | | Russian Federation, United States | | | Pakistan, China, Indonesia, India | | | |
| 1997 | India | | Russian Federation, United States | | | Pakistan, China, Indonesia, India | | | |
| 1998 | India | | Russian Federation, United States | | | Maldives, Guadeloupe | | | |
| 1999 | India | | Russian Federation, United States | | | Pakistan, India, Netherlands Antilles, Maldives, Guadeloupe | | | |
| Year | Grump | GPW | | GHS | | | LandScan | | WorldPop |
| 2000 | India | India, Maldives, Netherlands Antilles, Guadeloupe | | India, Pakistan, Netherlands Antilles, Maldives, Guadeloupe | | | India | | India |
| Year | GPW | | | | LandScan | | | WorldPop | |
| 2001 | India, Maldives, Netherlands Antilles, Guadeloupe | | | | India, Guadeloupe, Maldives, Netherlands Antilles | | | India | |
| 2002 | India | | | | India, Guadeloupe, Maldives, Netherlands Antilles | | | India | |
| 2003 | India | | | | India, Guadeloupe, Maldives, Netherlands Antilles | | | India | |
| 2004 | India | | | | India | | | India | |
| 2005 | India | | | | India,Guadeloupe,Maldives,Netherlands Antilles, Tuvalu | | | India | |
| 2006 | India, Guadeloupe, Maldives, Netherlands Antilles | | | | India,Guadeloupe,Maldives,Netherlands Antilles, Tuvalu | | | India | |
| 2007 | India, Guadeloupe, Maldives, Netherlands Antilles | | | | India | | | India | |
| 2008 | India, Netherlands Antilles, Maldives, Guadeloupe | | | | India,Guadeloupe,Maldives,Netherlands Antilles, Tuvalu | | | India | |
| 2009 | India, Netherlands Antilles, Maldives, Guadeloupe | | | | India,Guadeloupe,Maldives,Netherlands Antilles, Tuvalu | | | India | |
| 2010 | India, Netherlands Antilles, Maldives, Guadeloupe | | | | India,Guadeloupe,Maldives,Netherlands Antilles, Tuvalu | | | India | |
| 2011 | India, Maldives, Guadeloupe, Netherlands Antilles | | | | India | | | India | |
| 2012 | India, Maldives, Guadeloupe, Netherlands Antilles | | | | India | | | India | |
| 2013 | India, Maldives, Guadeloupe, Netherlands Antilles | | | | India | | | India | |
| 2014 | India, Maldives, Guadeloupe, Netherlands Antilles | | | | India | | | India | |
| 2015 | Guadeloupe, Maldives, Netherlands Antilles | | | | India | | | India | |
| 2016 | China, Democratic Republic of Congo, Maldives, Guadeloupe, Netherlands Antilles | | | | India | | | Guadeloupe, Maldives | |
| 2017 | China, Democratic Republic of Congo, Maldives, Guadeloupe, Netherlands Antilles | | | | India | | | Guadeloupe, Maldives | |
| 2018 | China, Democratic Republic of Congo, Guadeloupe | | | | Guadeloupe, Maldives | | | Guadeloupe, Maldives | |
| 2019 | Guadeloupe | | | | India | | | Guadeloupe, Maldives | |
| 2020 | Guadeloupe | | | | India, Guadeloupe, Maldives | | | Guadeloupe, Maldives | |

**Supplementary Table 3. The** **Coefficients of the quantile regression model for** **population prediction 1990-2020**

| Year | | Grump | | | | GPW | | GHS | |
| --- | --- | --- | --- | --- | --- | --- | --- | --- | --- |
| 1990 | | 0.473 | | | | 0 | | 0.528 | |
| 1991 | | 0.018 | | | | 0.04 | | 0.947 | |
| 1992 | | 0.088 | | | | 0.05 | | 0.867 | |
| 1993 | | 0.208 | | | | 0.039 | | 0.757 | |
| 1994 | | 0.451 | | | | 0.043 | | 0.541 | |
| 1995 | | 0.336 | | | | 0.012 | | 0.656 | |
| 1996 | | 0.133 | | | | 0.036 | | 0.847 | |
| 1997 | | 0.113 | | | | 0.063 | | 0.839 | |
| 1998 | | 0.067 | | | | 0.037 | | 0.902 | |
| 1999 | | 0.028 | | | | 0.049 | | 0.926 | |
| Year | | Grump | GPW | GHS | | | LandScan | | WorldPop |
| 2000 | | 0.235 | 0.043 | 0.357 | | | 0.283 | | 0.055 |
| Year | GPW | | | | LandScan | | | WorldPop | |
| 2001 | | 0.25 | | | 0.486 | | | 0.274 | |
| 2002 | | 0.056 | | | 0.519 | | | 0.434 | |
| 2003 | | 0.123 | | | 0.479 | | | 0.406 | |
| 2004 | | 0.159 | | | 0.446 | | | 0.402 | |
| 2005 | | 0.165 | | | 0.428 | | | 0.415 | |
| 2006 | | 0.024 | | | 0.388 | | | 0.600 | |
| 2007 | | 0.041 | | | 0.342 | | | 0.630 | |
| 2008 | | 0.336 | | | 0.336 | | | 0.332 | |
| 2009 | | 0.335 | | | 0.334 | | | 0.331 | |
| 2010 | | 0.007 | | | 0.476 | | | 0.532 | |
| 2011 | | 0.093 | | | 0.481 | | | 0.439 | |
| 2012 | | 0.048 | | | 0.470 | | | 0.497 | |
| 2013 | | 0.041 | | | 0.439 | | | 0.535 | |
| 2014 | | 0.013 | | | 0.450 | | | 0.553 | |
| 2015 | | 0.238 | | | 0.556 | | | 0.217 | |
| 2016 | | 0.072 | | | 0.841 | | | 0.107 | |
| 2017 | | 0.057 | | | 0.863 | | | 0.087 | |
| 2018 | | 0.038 | | | 0.836 | | | 0.126 | |
| 2019 | | 0.056 | | | 0.903 | | | 0.078 | |
| 2020 | | 0.076 | | | 0.921 | | | 0.023 | |

**Supplementary Table 4. The countries of census data that were excluded of model validation**

| SOC | Name | 1990 Population (Unit:k) |
| --- | --- | --- |
| BES | Bonaire, Sint Eustatius and Saba | 12.5 |
| BLM | Saint Barthélemy | 5.1 |
| CUW | Curaçao | 155.6 |
| FLK | Falkland Islands (Malvinas) | 2.3 |
| GGY | Guernsey | 57.4 |
| GUM | Guam | 137.0 |
| IMN | Isle of Man | 68.3 |
| JEY | Jersey | 82.6 |
| MYT | Mayotte | 89.9 |
| NIU | Niue | 2.5 |
| PSE | State of Palestine | 2,081.3 |
| SPM | Saint Pierre and Miquelon | 6.3 |
| TKL | Tokelau | 1.7 |
| VAT | Holy See | 0.7 |
| XKX | Kosovo (under UNSC res. 1244) | 1,947.3 |
